# Diagnostic Performance of Artificial Intelligence for Parkinson’s Disease Detection Using Handwriting and Drawing Tasks: A Systematic Review and Meta-Analysis

**DOI:** 10.3390/s26216893

**Published:** 2026-10-30

**Authors:** Philippe Gorce, Julien Jacquier-Bret

**Affiliations:** 1 Université de Toulon, CS 60584, CEDEX 9, 83041 Toulon, France; gorce@univ-tln.fr; 2 International Institute of Biomechanics and Occupational Ergonomics, Avenue du Docteur Marcel Armanet, CS 10121, CEDEX, 83418 Hyères, France

**Keywords:** deep learning, logit transformation, meta-analysis, sensors, risk of bias, publication bias, level of evidence, Parkinson-disease assessment, accuracy

## Abstract

This systematic review and meta-analysis aimed to evaluate the performance of deep learning (DL)-based methods for classifying individuals with Parkinson’s disease (PD) from handwriting and drawing tasks, following PRISMA guidelines. Google Scholar, IEEE Xplore, PubMed/MEDLINE, ScienceDirect, and Science.gov were searched from 1 July to 17 July 2026, without publication date restrictions. Eligible studies evaluated a DL architecture for PD detection using handwriting or drawing and reported at least one of five performance metrics: accuracy, sensitivity, specificity, precision, or F1-score. Two reviewers independently performed study selection, data extraction, and risk-of-bias assessment using PROBAST+AI. Performance estimates were logit-transformed and pooled separately using random-effects models. Heterogeneity was assessed using Cochran’s Q, I^2^, and τ^2^, and results were presented using forest plots with 95% confidence intervals. Potential sources of heterogeneity were investigated through subgroup analyses according to DL architecture, dataset, task, and validation procedure, together with sensitivity analyses. Publication bias was assessed using Egger’s test, and certainty of evidence was evaluated using GRADE. Of 5233 unique records identified, 30 studies were included. Overall pooled performance exceeded 92% across the evaluated metrics. No significant differences were found between fully DL and hybrid approaches or among DL algorithms. Dataset-related differences were observed but were inconsistent across performance metrics. Combined handwriting and drawing tasks consistently achieved higher performance (>95%) than individual tasks, while cross-validation was associated with higher accuracy, specificity, and sensitivity than hold-out validation. Despite these promising findings, substantial heterogeneity and very low certainty of evidence limit the current clinical applicability of these approaches. More standardized protocols, subject-level data separation, and external validation in independent populations are needed to develop robust and generalizable AI models and clarify the potential of handwriting and drawing as non-invasive tools for PD detection.

## 1. Introduction

Artificial intelligence (AI) methods based on a deep learning (DL) approach represent a promising and scientifically dynamic approach to identifying the presence of Parkinson’s disease (PD). PD is one of the most common disease worldwide [1] and has the fastest-growing prevalence [2]. Between 1990 and 2021, the number of people affected quadrupled [3], reaching nearly 12 million. Su et al. projected that more than 25 million people worldwide will be affected by 2050 [4]. PD is multifactorial and manifests through motor (e.g., bradykinesia, tremor, postural instability [5,6]) and neurological symptoms (e.g., anxiety, depression, cognitive impairment [7]). This progressive disease, whose symptom severity varies from one individual to another, initially manifests as difficulties performing daily activities and can progress to a complete loss of autonomy. In this context, early diagnosis is a major area of research aimed at optimizing therapeutic management [8].

Several studies have developed AI methods using DL architectures to classify PD patients versus healthy controls (HC) based on the analysis of tremor [9], gait [10], or walking [11]. Other studies have used handwriting and drawing to distinguish PD patients from HC. These complex fine-motor activities require the coordinated integration of range of motion, velocity, timing, pressure, and visuomotor control—all of which are susceptible to impairment by PD. Changes in one or more of these parameters can thus provide discriminating information even when motor deficits are relatively mild [12,13]. Wearable and non-wearable technological advances, particularly digital tablets and image acquisition systems, have significantly expanded the range of diagnostic information that can be extracted from handwriting and drawings. In addition to static spatial characteristics derived from the final image, digital capture allows for the assessment of dynamic and kinematic parameters such as the trajectory of the pen tip, velocity, acceleration, pressure, drawing duration, and movements made in the air [14,15]. The use of DL-based AI has automated the identification of complex patterns associated with PD from handwriting and drawings. Pereira et al. demonstrated the feasibility of using convolutional neural networks (CNN) to identify PD based on handwriting dynamics [16], and subsequent studies have extended DL approaches to various handwriting and drawing datasets [17,18]. Consequently, combining writing and drawing tasks with DL-based classification represents a promising, objective, noninvasive, and potentially scalable approach for distinguishing PD patients. Such methods could, in the long term, contribute to computer-assisted screening and facilitate the identification of subtle graphomotor abnormalities associated with the early stages of the disease.

To achieve this, it is necessary to ensure that these methods demonstrate optimal detection performance that can be generalized to all affected individuals in a real-world evaluation setting. Performance is most often evaluated using several metrics, the most commonly used being accuracy, precision, specificity, sensitivity, and the F1 score [19,20]. These metrics enable several aspects of classification to be considered, particularly classification errors related to false positives and false negatives. Several studies have reported high detection performance for PD [21,22,23]. However, it is difficult to directly compare these performance metrics since the DL architectures used differ (CNN, recurrent neural networks-RNN, transformers, etc.), as do the datasets and tasks studied. The use of systematic reviews with meta-analyses allows these methodological differences among AI methods to be accounted for and enables the exploitation of performance results by category, particularly through subgroup analyses [24,25,26].

To the best of our knowledge, no systematic review and meta-analysis has specifically assessed the performance of DL-based methods for PD detection using handwriting and drawing tasks. Therefore, this study aimed to systematically synthesize and quantitatively assess the performance of DL approaches reported in the literature. The review was conducted in accordance with the PRISMA guidelines (Preferred Reporting Items for Systematic Reviews and Meta-Analyses [27]) to ensure transparent and rigorous reporting of the review process. In addition, subgroup analyses were performed to investigate potential sources of heterogeneity across five standard performance metrics, focusing on four methodological factors: DL architecture, dataset, handwriting or drawing task, and validation procedure. Since the algorithms aim for maximum detection, an asymmetry in the values emerges that must be corrected through a transformation to stabilize the variance and satisfy the normality assumptions of the random-effects models used in the meta-analysis. This issue has been addressed in previous studies [28,29], and a recent meta-analysis demonstrated, through comparison, that the most appropriate transformation in the AI context is the logit transformation [26]. This approach was applied to provide evidence for improving the early detection of PD and, consequently, patient care.

## 2. Materials and Methods

### 2.1. Study Design

This study is a systematic review with a meta-analysis conducted in accordance with the PRISMA guidelines [27,30,31]. The full protocol has been registered in the International Prospective Register of Systematic Reviews (PROSPERO) [32] under number CRD420261497673.

### 2.2. Search Strategy

Five electronic databases were searched without restrictions on publication date: Google Scholar, IEEE Xplore, PubMed/MEDLINE, ScienceDirect, and Science.gov. The searches were performed from 1 to 17 July 2026 using database-specific combinations of keywords connected by the Boolean operators AND and OR. The search strategy was adapted to the requirements of each search engine; in particular, the ScienceDirect query was modified to meet its eight-Boolean-operator limit. The complete search strings applied to each database are reported in Table 1.

### 2.3. Selection Process

All records retrieved from the five databases were merged into a single Microsoft Excel file, where duplicate entries were identified and removed using an automated function. Study eligibility was subsequently assessed in two stages by two independent reviewers. First, titles and abstracts of the remaining unique records were screened according to the predefined eligibility criteria. The reviewers’ decisions were then compared, and disagreements were discussed until consensus was reached. Articles retained at this stage underwent independent full-text assessment by both reviewers using the same inclusion and exclusion criteria. Studies failing to meet the eligibility requirements after full-text examination were excluded. The reviewers then compared their final decisions to determine the studies included in the systematic review, with any remaining disagreement resolved through discussion and reassessment of the corresponding article. Inter-reviewer agreement was high (Cohen’s κ = 0.87).

### 2.4. Selection Criteria

The kinematic and dynamic characteristics of writing and drawing activities can be used to identify PD patients. The latest sensor technologies, such as digital tablets, touchscreens, or digital pens equipped with sensors, enable the collection of geometric information about the stroke and task-related data such as spatial coordinates, pressure, time, velocity, acceleration, and air movements, where applicable. This data is then processed into images or temporal/kinematic features and used as input for DL models to distinguish PD patients from healthy control subjects.

Then, to be included, a study had to examine at least one handwriting task such as drawing circles, meanders, spirals, or writing letters, words, or sentences using wearable or not wearable sensors. The AI method used had to be based on a DL approach that could distinguish PD patients from healthy controls. The method’s classification performance had to be evaluated using at least one of the five commonly used metrics: accuracy, specificity, sensitivity, precision, and F1 score. The studies also had to provide the necessary information (confusion matrix or number of features used by the model) to apply the logit transformation to the performance metrics (see below, Section 2.7). Only original, peer-reviewed studies published in English were considered.

Several criteria led to the exclusion of a study: (1) the format was that of a conference paper, a book or book chapter, a literature review, a report, a case study, etc.; (2) the study was not written in English and peer-reviewed; (3) the classification was not based on at least one handwritten writing task or was not evaluated using performance metrics; (4) the method was not sufficiently detailed to classify the algorithms or perform the logit transformation of the performance metrics.

### 2.5. Risk of Bias Assessment

PROBAST+AI (Prediction Model Risk of Bias Assessment Tool + Artificial Intelligence) was used to examine the methodological rigor and applicability of the included studies [33]. The tool separately considers model development, focusing on methodological quality, and model evaluation, addressing potential risk of bias in the reported performance estimates. Both components cover four domains—Participants and Data Sources, Predictors, Outcome, and Analysis—and include 16 and 18 signaling questions, respectively. Signaling questions were answered as “Yes”, “Probably yes”, “Probably no”, “No”, “No information”, or “Not applicable”, where appropriate. Responses of “Yes” and “Probably yes” generally supported adequate methodological conduct or a low risk of bias, whereas “No” and “Probably no” indicated potential methodological concerns. Considering the signaling questions together with a reasoned methodological judgment, each domain was classified as “Low”, “High”, or “Unclear”. Overall judgments were subsequently determined separately for model development quality and model evaluation risk of bias. Applicability to the review question was examined for the Participants and Data Sources, Predictors, and Outcome domains and categorized as “Low”, “High”, or “Unclear” concern. For studies addressing both model development and model evaluation, each PROBAST+AI component was assessed independently. Two reviewers (J.J.B. and P.G.) performed all assessments separately, with disagreements resolved through discussion and consensus. Study-level judgments for the evaluation component were displayed using a traffic-light plot, while the remaining PROBAST+AI assessments were reported in a summarized format [34].

### 2.6. Data Extraction and Classification

For each included study, data extraction covered bibliographic, methodological, and performance-related information. Bibliographic characteristics included the first author, publication year, and country. Methodological information comprised the AI approach (DL or hybrid DL–machine learning (ML)), the model family and specific algorithms used (e.g., CNN, RNN, artificial neural network (ANN), and long short-term memory (LSTM), etc), the dataset, the number and distribution of PD participants and HC, the handwriting or drawing tasks, and the validation strategy. The number of events and all available values for accuracy, specificity, sensitivity, precision, and F1-score were also collected. These performance metrics were defined from the four outcomes of PD versus HC classification: true positives (TP), representing correctly identified PD cases; true negatives (TN), correctly identified HC; false positives (FP), HC incorrectly classified as PD; and false negatives (FN), PD cases incorrectly classified as HC. Accordingly, the metrics were calculated as follows: accuracy = (TP + TN)/(TP + TN + FP + FN), specificity = TN/(FP + TN), sensitivity = TP/(TP + FN), precision = TP/(TP + FP), and F1-score = 2 × (sensitivity × precision)/(sensitivity + precision).

When multiple models or algorithms were evaluated on the same dataset within a single study, only the solution with the best performance was selected for extraction and meta-analysis. This approach was intended to avoid the duplication of non-independent observations from the same population and the overrepresentation of studies comparing a large number of models. When multiple datasets were evaluated within a single study, the best solution was selected separately for each dataset.

### 2.7. Data and Statistical Analysis

Given the high heterogeneity in performance observed in previous meta-analyses [35,36], a random-effects model estimated using the restricted maximum likelihood (REML) method was selected. This model assumes an approximately normal distribution of the observed effects around their true effects, as well as of the true effects across studies. However, the performance metrics under study are proportions bounded between 0 and 1, and when their values are close to these bounds, their distribution may be skewed and their variance unstable. A transformation of the proportions prior to their aggregation is therefore recommended in order to stabilize their variance and bring their distribution closer to normality [37], thereby ensuring a better fit to the assumptions of the random-effects model estimated by REML. The logit transformation maps the proportions from the bounded interval [0, 1] to the real line [38]. It is being used more and more because it reduces asymmetry and supports the application of random-effects models. A recent study conducted in the context of posture recognition for the prevention of work-related musculoskeletal disorders in occupational activities showed that the logit transformation yielded the best results for evaluating the performance of AI methods compared to the Freeman-Tukey transformation or untransformed data [26]. The logit transformation was therefore applied to the five performance metrics prior to pooling, according to the following formula:

$$\mathrm{logit}\left(p\right)=\;\ln\left(\frac{p}{1-p}\right)=\alpha$$

where *p* is the performance proportion (between 0 et 1), and *ln* is the natural logarithm. When *p* = 0 or *p* = 1, the logit transformation is undefined. In this case, a Haldane-Anscombe continuity correction (*p*_(*HA*)_) [39,40] was applied to recompute *p* before performing the logit transformation:

$$p_{\left(\mathrm{HA}\right)}=\;\frac{x+0.5}{n+1}$$

where *x* is the number of favorable cases and *n* is the sample size. The corresponding standard error (*SE*) was computed as follows:

$$\left\{\begin{array}{c} {\mathrm{SE}}_{\mathrm{logit}}\left(p\right)=\;\sqrt{\frac{1}{x}+\frac{1}{n-x}}\mathrm{if}\;0<p<1\\ {\mathrm{SE}}_{\mathrm{logit}}\left(p\right)=\;\sqrt{\frac{1}{x+0.5}+\frac{1}{n-x+0.5}}\mathrm{if}\;p=0\;\mathrm{or}\;1 \end{array}\right.$$

where *x* is the number of favorable cases and *n* is the sample size.

Separate meta-analyses were performed for each performance metric. Heterogeneity was assessed using Cochran’s Q test (*p* < 0.10) and I^2^ [41], categorized as low (<25%), moderate (25–50%), substantial (50–75%), or high (>75%). Between-study variance (τ^2^) was estimated using REML under a random-effects model with inverse-variance weighting. Pooled estimates and 95% confidence intervals were presented in forest plots.

Five subgroup analyses examined potential sources of heterogeneity according to DL approach (fully DL vs. hybrid DL–ML), DL architecture, dataset, handwriting or drawing task, and validation strategy. Differences between subgroup categories were tested using pairwise mixed-effects meta-regressions. Robustness was assessed through two sensitivity analyses: leave-one-out, sequentially excluding each algorithm, and leave-one-study-out, simultaneously excluding all estimates from each study. Finally, publication bias was evaluated by visual inspection of funnel-plot asymmetry and Egger’s regression test [42].

To interpret and discuss the results, all logit-transformed performance values were back transformed (*pBT*) to their original scale (between 0 and 1) using the following equations:

$${\mathrm{pBT}}_{\mathrm{logit}}=\;\frac{e^{\alpha }}{1+e^{\alpha }}$$

where *α* represents the value on the logit scale.

All analyses were performed using R (R Core Team, v4.6.0, Vienna, Austria) and JASP (JASP Team, v0.96, Amsterdam, The Netherlands) software with a significance threshold set at 5%.

### 2.8. Certainty of Evidence

Evidence certainty was assessed using the GRADE (Grading of Recommendations Assessment, Development, and Evaluation) framework [43] across five domains: risk of bias, indirectness, inconsistency, imprecision, and publication bias. Each domain was judged as presenting either serious or non-serious concerns. Starting from high certainty, the rating was downgraded by one level for every serious concern, resulting in a final classification of high, moderate, low, or very low certainty. Two reviewers (P.G. and J.J.B.) independently performed the assessment and resolved discrepancies through consensus.

## 3. Results

### 3.1. Search Results

**Figure 1:**
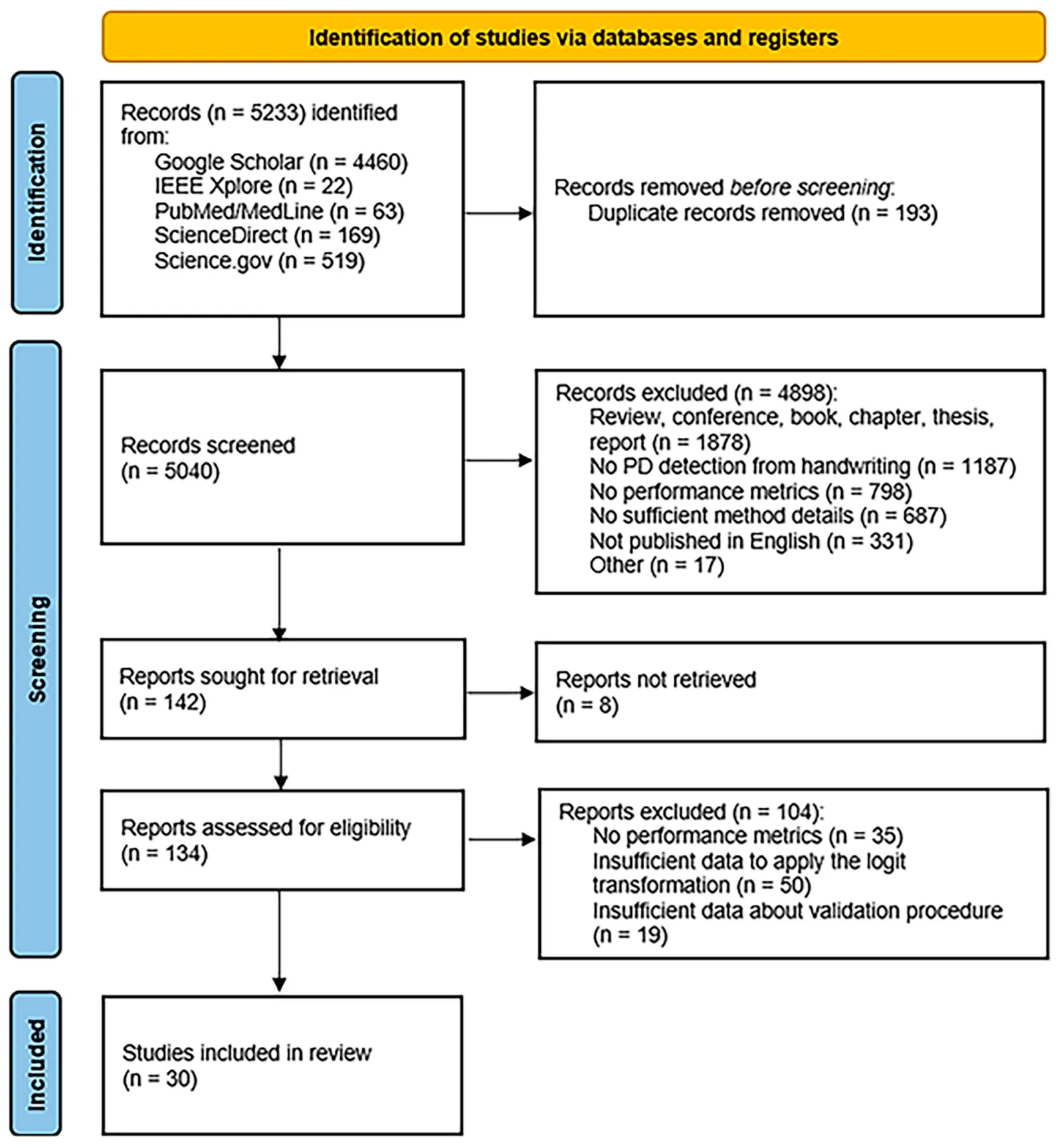
PRISMA flow diagram.

A search of the five databases identified 5233 articles. After removing 193 duplicates, 4898 articles were excluded based on their titles and abstracts in accordance with the inclusion and exclusion criteria. The main reasons for exclusion were formats other than original research (reviews, books or book chapters, conference papers, reports, etc.), PD detection not based on handwriting tasks, the absence of expected performance metrics, or a lack of details regarding the method used, making it impossible to form subgroups. From the remaining 142, 104 were excluded after reading the full text because performance metrics were missing or not directly related to a handwriting task, data enabling logit transformation were absent, or method validation was missing or insufficiently detailed. Thus, 30 original research articles were included in the meta-analysis. The selection process is detailed in Figure 1.

### 3.2. Study Characteristics

Table 2 presents the full characteristics of the 30 included studies. Fourteen countries are represented [16,17,21,22,23,44,45,46,47,48,49,50,51,52,53,54,55,56,57,58,59,60,61,62,63,64,65,66,67,68]. Twenty-four studies proposed a method based solely on a DL approach [16,17,21,22,23,44,45,46,47,48,49,50,51,53,54,55,56,57,58,61,65,66,67,68], while the other 6 proposed a hybrid approach in which the DL algorithm was combined with an ML classifier [52,59,60,62,63,64].

CNN-based methods largely predominated, with a CNN component present in 25 of the 30 approaches (83.3%), either as a standalone architecture (56.7%) or combined with other components (26.7%) [16,17,22,44,46,47,48,49,50,51,52,53,54,55,56,57,58,59,60,61,62,64,65,67,68]. Transformer-based models accounted for 10.0% of approaches [21,45,66], while ANN and RNN architectures were each represented once [23,63]. Some approaches also combined DL-based feature extraction with conventional ML classifiers, mainly SVM and KNN.

The included studies used 11 different datasets, predominantly NewHandPD (17 studies), HandPD (7 studies), and PaHaW (7 studies), with several studies evaluating more than one dataset. Drawing was the predominant task, particularly spiral drawing, which was assessed in all 30 studies, followed by meander, circle, and wave tasks. Handwriting was less frequently investigated (6 studies) and included letters, numbers, words, and sentences.

Hold-out validation was the most common strategy (18 studies; 60.0%), using various train–test splits, whereas 12 studies (40.0%) applied cross-validation with different configurations. Notably, only four studies used subject-wise procedures, including 5- or 10-fold subject-wise CV and LOSO-CV [49,63,67,68]. Among studies using hold-out validation, only two reported a separate validation set [58,66].

As summarized in Table 3, accuracy was reported in all 30 studies, followed by sensitivity (*n* = 21), precision (*n* = 14), F1-score (*n* = 13), and specificity (*n* = 12). Only four studies reported all five performance metrics [45,57,59,64], highlighting substantial variability in performance reporting across the included studies.

### 3.3. Risk of Bias

**Figure 2:**
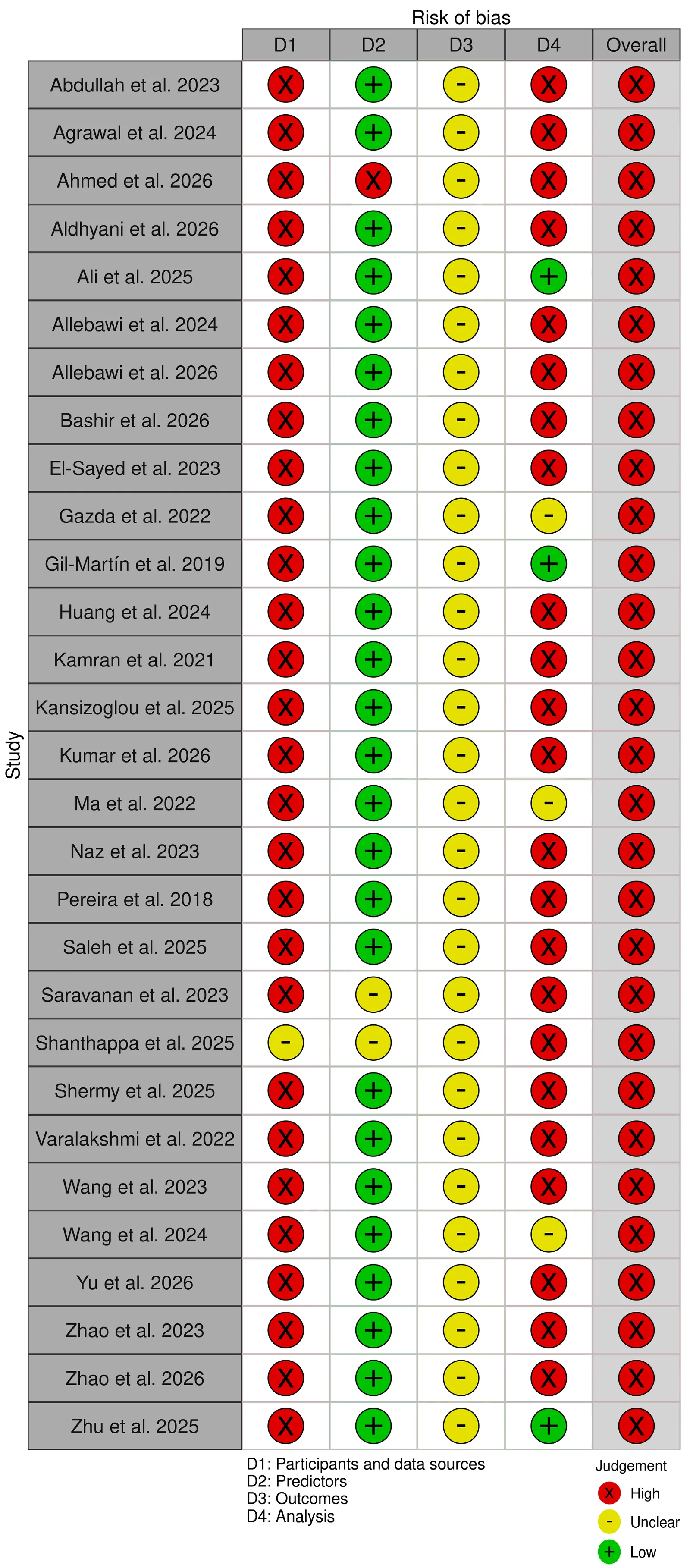
Traffic light plot illustrating the risk of bias of the evaluation section of the PROBAST+AI. References [16,17,21,22,23,44,45,46,47,48,49,50,51,52,53,54,55,56,57,58,59,60,61,62,63,64,65,66,67,68].

**Figure 3:**
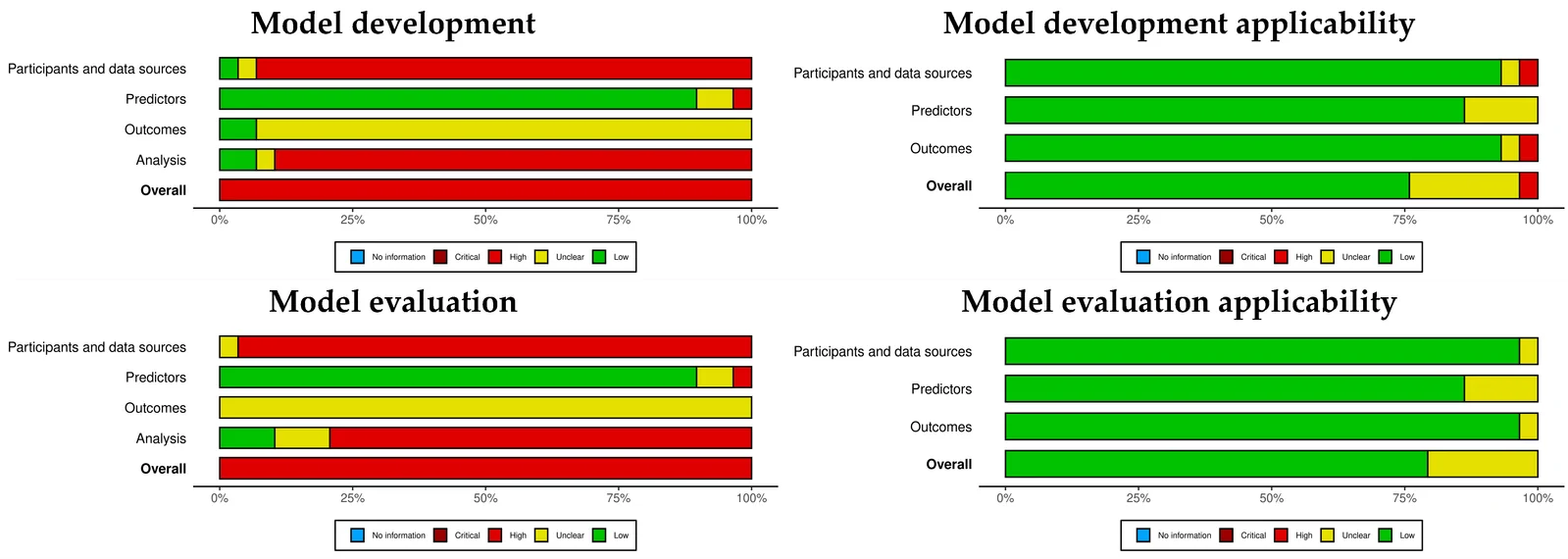
Summary of the risk of bias assessment for the development, evaluation and applicability of the models proposed in the studies included from PROBAST+AI.

The PROBAST+AI tool was used to assess the quality of the selected studies. The results showed good applicability of the models (Figure 2 and Figure 3). However, a high risk of bias was observed for domains D1 “Participants and Data Sources” and D4 “Analysis” in the majority of included studies, both during development and evaluation. Specifically, for domain D1, the models are based on datasets with small sample sizes, sometimes unbalanced between PD and HC participants, with differences in sample sizes between men and women, or in the age of subjects within the groups. Furthermore, some datasets contain multiple trials for the same subject, with a lack of detail regarding the actual number of subjects included. These elements reduced the population representativeness. For domain 4, the risks stem primarily from the fact that validations are almost never external, i.e., on an independent cohort, and the absence of explicit subject-wise data splitting despite repeated observations per participant, creating potential data leakage and optimistic performance estimates. These two domains explain why all the included studies presented an overall high risk of bias for development and evaluation.

### 3.4. Meta-Analysis—Overall Performance Metrics

Overall performance exceeded 90% (Table 4). Specificity was the parameter with the lowest performance (91.62%), and precision was the one with the highest value (95.76%). However, heterogeneity varied across the parameters. Moderate heterogeneity was observed for precision, substantial heterogeneity for sensitivity and F1-score, and high heterogeneity for accuracy and specificity. The estimated between-study variance on the logit scale ranged from τ^2^ = 0.41 for precision to τ^2^ = 0.95 for specificity (see Supplementary Files S1–S5 for the results of the forest plots).

### 3.5. Exploration of Possible Causes of Heterogeneity—Subgroup Analysis

#### 3.5.1. DL vs. Hybrid

Overall performance exceeded 90% for both exclusively DL and hybrid methods (Table 5). No significant difference was observed between the two AI methods after the pairwise mixed-effects meta-regression. For sensitivity, precision, and F1-score, a significant reduction in heterogeneity of 20 to 35% was observed for DL methods compared to hybrid methods.

#### 3.5.2. Algorithmic Architecture of DL Models

All pooled performance metrics showed performance values above 90% (Table 6). Only the accuracy of CNNs exhibited high heterogeneity (I^2^ > 85%). All performance metrics for transformers showed low heterogeneity (I^2^ = 0%). Heterogeneity for the other parameters was mostly rated as moderate, with the exception of CNNs and combined CNNs for specificity (substantial heterogeneity). Pairwise mixed-effects meta-regressions revealed a significant difference in precision, with a lower value for the CNN algorithm (93.48%) compared to a combined CNN architecture (96.99%, *p* < 0.05) (see Appendix A for details on all pairwise mixed-effects meta-regressions).

#### 3.5.3. Datasets

All performance metrics grouped by dataset achieved a performance of over 90%, except for accuracy on the PaHaw dataset (88.09%) and specificity on the HandPD dataset (83.88%, Table 7). Variations in heterogeneity were observed depending on the dataset and the performance metric. The HandPD dataset exhibited heterogeneity ranging from 50 to 68% for precision, sensitivity, and accuracy, and less than 30% for the other two metrics. The PaHaW dataset showed high heterogeneity for precision and specificity, and low heterogeneity for the other indicators. NewHandPD was the dataset with the highest heterogeneity. Paired mixed-effects meta-regressions showed that the choice of dataset significantly influenced precision. PaHaW had the lowest accuracy (88.09%), which was significantly different from the Combined and NewHandPD datasets (*p* < 0.05), while “Parkinson’s Drawing” yielded a higher accuracy (96.59%) than HandPD and PaHaW (*p* < 0.05). No differences were observed for the other performance metrics (*p* > 0.05) (see Appendix B for details on all pairwise mixed-effects meta-regressions).

#### 3.5.4. Drawing vs. Handwriting

Subgroup analyses showed generally high pooled performance across tasks. Combined tasks consistently yielded the highest pooled estimates, with an accuracy of 96.23% (95% CI: 94.87–97.23%), specificity of 95.61% (91.74–97.71%), sensitivity of 95.97% (93.72–97.44%), precision of 97.24% (95.65–98.25%), and F1-score of 97.54% (96.17–98.43%, Table 8). Among individual tasks, spiral and meander achieved generally showed high performance (>92%), whereas circle and wave tasks tended to yield lower estimates (<90% pour accuracy, specificity and sensitivity). Handwriting also showed high sensitivity (94.44%), precision (94.99%), and F1-score (94.25%) (see Appendix C for details on all pairwise mixed-effects meta-regressions).

#### 3.5.5. Comparison of Validation Procedure

All performance metrics achieved values above 90%. Heterogeneity was substantial (ranging from 50 to 75%) for all metrics, regardless of the validation method. Only the precision and F1-score from the hold-out validation showed moderate heterogeneity (ranging from 25 to 50%). Paired mixed-effects meta-regressions revealed higher accuracy, specificity, and sensitivity with cross-validation compared to hold-out with train/test validation (*p* < 0.05, Table 9).

### 3.6. Sensitivity Analysis

Sensitivity analyses demonstrated excellent robustness of the results for all performance metrics. Successively excluding algorithms one by one resulted in changes in the pooled values of 0.5% or less (Table 10). Excluding the studies that produced these largest differences led to a reduction in heterogeneity, particularly for sensitivity (−5%), precision (−12%), and F1-score (−10%).

In the leave-one-study-out analysis, the pooled performance variations were less than 1.5% (Table 11). The largest variation was observed for specificity (1.45%) and the smallest for sensitivity (0.47%). As in the previous analysis, excluding the studies that caused the greatest variation resulted in a 10% reduction in heterogeneity (I^2^) for four of the metrics (accuracy, specificity, precision, F1-score).

### 3.7. Publication Bias

**Figure 4:**
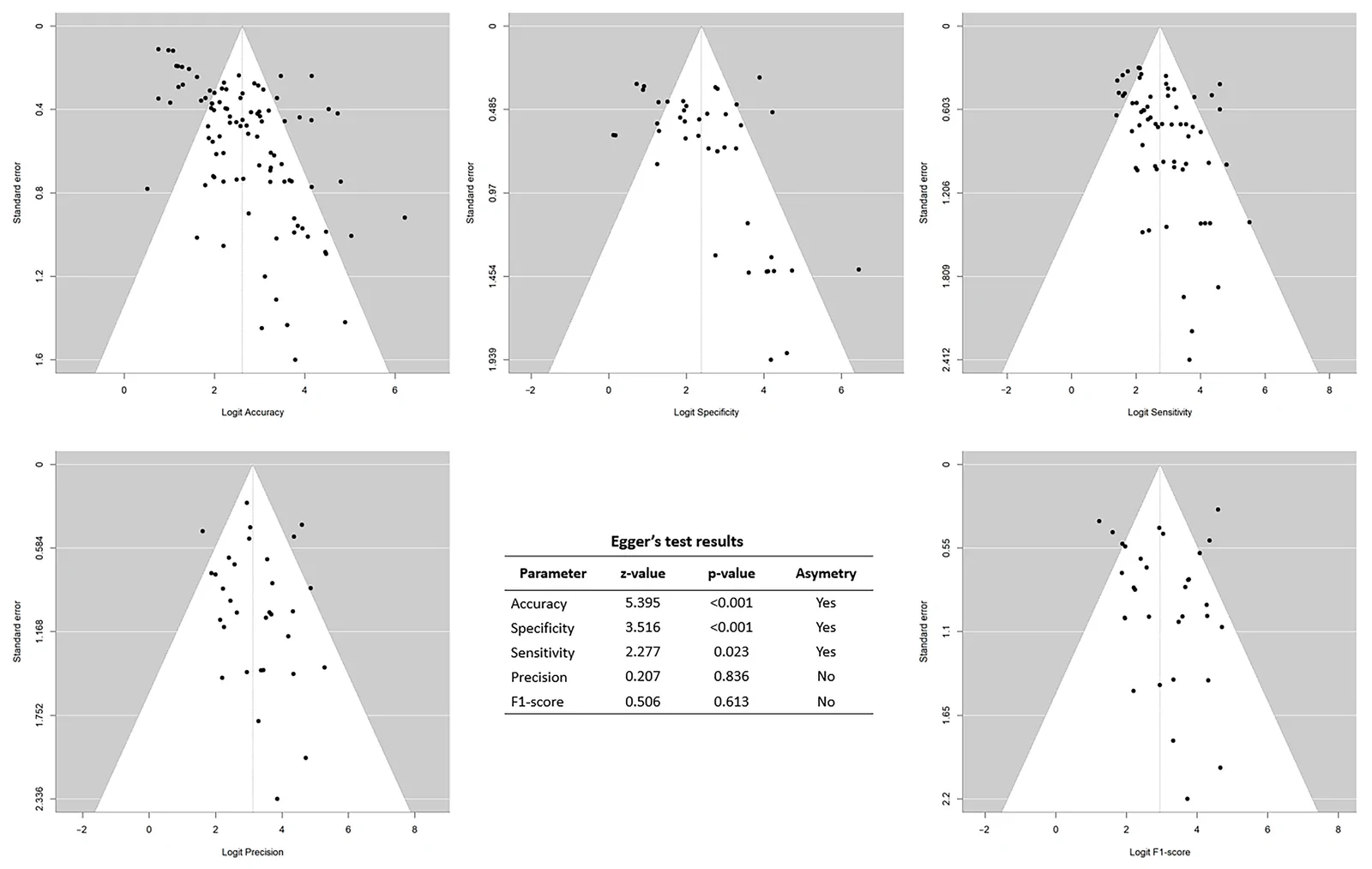
Funnel plots and Egger’s test results for each performance parameter.

Egger’s regression test indicated significant funnel plot asymmetry for accuracy (z = 5.40, *p* < 0.001), specificity (z = 3.52, *p* < 0.001), and sensitivity (z = 2.28, *p* = 0.023). In contrast, no significant evidence of funnel plot asymmetry was observed for precision (z = 0.21, *p* = 0.836) or F1-score (z = 0.51, *p* = 0.613, Figure 4). These findings suggest the presence of publication bias or small-study effects for accuracy, specificity, and sensitivity, whereas no such evidence was identified for precision or F1-score.

### 3.8. Level of Evidence

The overall certainty of the evidence was assessed using the GRADE framework. The certainty of the evidence was judged to be very low for accuracy, specificity, sensitivity, and the F1 score, while precision was classified as having low certainty (Table 12). The downgrade from the “high” level of certainty in the diagnostic test accuracy studies is primarily due to serious concerns regarding risk of bias, indirectness, and inconsistency. These results indicate that the seemingly high diagnostic performance of AI methods for PD detection based on handwriting and drawing tasks should be interpreted with caution.

## 4. Discussion

The objective of this systematic review with meta-analysis was to provide an overview of the capabilities of AI solutions using a DL approach to detect PD based on handwriting and drawing tasks. The performance of the best proposed solutions, based on 30 studies, was evaluated by considering various parameters: the DL model architecture, the dataset used, the type of task studied, and the validation method. This review is part of an effort toward early diagnosis of PD to improve patient care.

### 4.1. Performance Results for PD Detection

The results showed that DL-based AI approaches achieved high performance for PD detection from handwriting and drawing, with all pooled metrics exceeding 90% and precision and F1-score reaching approximately 95%. Sensitivity is particularly important in a healthcare context because it reflects the ability to correctly identify patients with PD. The pooled sensitivity of 93.93% indicates that only about 6% of PD cases remained undetected. Further improving sensitivity toward 100% would therefore be desirable to minimize missed cases and facilitate early clinical management, in accordance with recommendations for medical AI systems [69,70]. Classification errors nevertheless remained limited overall. The high precision (95.76%) indicates that individuals identified as having PD were rarely false positives, whereas the lower specificity (91.62%) suggests a greater tendency to misclassify healthy individuals as patients. Such false positives may partly arise from the limited specificity of graphomotor abnormalities to PD, as micrographia and tremor-related alterations can also occur in essential tremor, dystonia, and atypical parkinsonian disorders [14,71]. Models trained exclusively on PD patients and healthy controls may therefore detect graphomotor impairment rather than PD-specific patterns, potentially generating false positives in patients with other neurological disorders. These errors should also be considered according to their clinical consequences. False positives may cause patient anxiety, lead to unnecessary additional examinations, and, when frequent, reduce confidence in the system [72]. However, when AI is used as a screening or decision-support tool rather than as a standalone diagnostic method, false-positive classifications can subsequently be reassessed through clinical evaluation. Conversely, false negatives are potentially more concerning because affected patients may remain undetected. The high sensitivity observed in this meta-analysis is therefore encouraging for the use of handwriting- and drawing-based AI as a potential screening tool complementing clinical diagnosis.

Nevertheless, these results should be interpreted cautiously given the low to very low certainty of evidence. GRADE identified concerns related to inconsistency and indirectness, notably because of small and frequently imbalanced datasets and the lack of external validation, which limit the generalizability of the findings.

### 4.2. Exploration of Causes of Heterogeneity—Subgroup Analyses

Substantial and high heterogeneity was observed among the five performance parameters. Subgroup analyses allow the exploration of various potential sources that might explain the differences across studies [73] through the following comparisons: DL architecture, datasets, tasks, validation process.

#### 4.2.1. DL Architecture

The first subgroup analysis showed no significant differences between fully DL and hybrid DL–ML approaches for any performance metric, consistent with previous meta-analyses in breast cancer [74] and lung cancer [75]. These findings suggest that replacing the final DL classification layers with a conventional ML classifier does not necessarily improve performance when discriminative features have already been learned by the deep network. Similarly, pairwise mixed-effects meta-regressions showed no significant differences between CNN, combined CNN, and Transformer architectures for most metrics, except for higher precision with combined CNN than conventional CNN. Overall, no DL architecture consistently outperformed the others, in agreement with previous meta-analyses on COVID-19 severity [76] and ovarian cancer classification [77]. Together, these findings suggest that model architecture may not be the primary determinant of classification performance.

#### 4.2.2. Datasets and Tasks

Unlike the model architecture, the choice of dataset appeared to have a more pronounced influence on the reported classification performance. Several significant differences were observed across the datasets. However, these differences were not systematic, suggesting that performance may depend in part on characteristics specific to the datasets rather than solely on the model’s architecture.

Such variability could be explained by differences in sample size, class distribution, participant characteristics, or disease severity [78]. Other parameters, more closely related to study protocols—such as data acquisition devices or the type and number of handwriting and drawing tasks included—could also account for the heterogeneity of the observed differences. Indeed, all these factors are likely to alter the nature and complexity of the input data and, consequently, influence the apparent performance of AI models [79]. Furthermore, development datasets generalize poorly to other datasets, particularly during external validation. Based on 83 studies and 86 DL algorithms, Yu et al. [80] showed that external validation led to a decline in performance in 81% of cases.

Consequently, the high performance reported for a given algorithm may not reflect solely the superiority of the model itself, but may also depend significantly on the dataset on which it was developed and evaluated.

#### 4.2.3. Tasks

Dataset choice showed several significant associations with performance, although these were inconsistent across metrics. Differences in sample size, class distribution, participant characteristics, and disease severity [78], as well as acquisition protocols and graphomotor tasks, may contribute to this variability [79]. Dataset dependence also raises concerns regarding generalizability: Yu et al. [80] reported decreased performance during external validation in 81% of evaluated DL algorithms. Thus, high reported performance may reflect both model characteristics and the dataset on which it was developed and evaluated. Task type was also associated with performance. Despite its widespread use [81], spiral drawing showed no systematic superiority over other individual tasks. In contrast, combined tasks generally performed better, particularly for accuracy, potentially because different graphomotor activities provide complementary information [82]. As in the present study, a systematic review by Huang et al. [83] reported improved accuracy when medical imaging was combined with clinical data, compared to unimodal models. Similar benefits associated with the integration of multimodal data have also been reported in other medical AI applications [84,85]. However, the benefit of combining multiple sources of information likely depends on the extent to which each source provides complementary rather than redundant information.

#### 4.2.4. Validation Procedure

Cross-validation produced significantly higher accuracy, specificity, and sensitivity than hold-out validation, with no differences in precision or F1-score. This may partly result from using more available data for training and reducing dependence on a single train–test split, which is particularly relevant for small datasets [86].

However, higher cross-validation performance does not necessarily indicate better generalizability. When multiple samples originate from the same participant, sample-level splitting can introduce information leakage if data from one individual occur in both training and testing sets [87]. This issue can affect both cross-validation and hold-out procedures. As only four studies explicitly reported subject-wise validation, differences between validation strategies may also reflect study design and data distribution. Participant-level separation is therefore essential for the reliable assessment of generalization to unseen individuals [88].

#### 4.2.5. Heterogeneity and Level of Evidence

Despite subgroup analyses, heterogeneity remained substantial or high for most outcomes, suggesting that it arises from multiple interacting methodological factors rather than a single source. Model architecture, datasets, graphomotor tasks, input features, and validation procedures may jointly contribute to this variability. Future studies with sufficient data could use multivariable meta-regression to assess several moderators simultaneously [89] and multilevel meta-analysis to account for dependent AI solutions within individual studies [90]. Such analyses were not possible here because the limited number of studies relative to the number of potential moderators would have increased model instability and overparameterization.

Finally, pooled estimates and subgroup effects should be interpreted cautiously because GRADE indicated very low certainty of evidence. More rigorous and standardized study designs are therefore needed. PROBAST+AI may help identify and prevent methodological biases [91], which were high across the included studies, particularly because of limited population representativeness and weaknesses in validation procedures. More robust internal validation and independent external validation are essential to obtain reliable estimates of performance and generalizability before clinical implementation.

### 4.3. Practical Outcomes and Future Research Directions

Despite the overall high classification performance, the PROBAST+AI and GRADE evaluation highlighted a significant risk of bias and low level of evidence, calling for caution regarding the generalizability of the results and their clinical application. Future studies should include sufficiently large samples that are representative of the demographic and clinical diversity of patients with Parkinson’s disease in order to improve generalizability to new patients [92]. Writing and drawing tasks, as well as the conditions under which they are performed, should also be clearly described and standardized.

Particular attention should be paid to validation procedures. Splitting the data at the participant level allows performance to be evaluated on individuals not encountered during training and thus provides a more relevant estimate of generalizability [93]. External validation on independent populations and under independent acquisition conditions should also be prioritized to assess the actual transferability of the models [94,95], as performance metrics derived solely from internal validations may be overly optimistic [96].

Finally, the observed heterogeneity underscores the importance of better standardization of protocols. The characteristics of datasets, acquisition methods, and validation procedures can directly influence model performance [78]. The use of common benchmark datasets and standardized evaluation protocols would thus facilitate a more objective comparison of different AI approaches [8].

### 4.4. Limitations

The first limitation concerns the objective of the classification, which was to distinguish between PD patients and healthy subjects. It would be interesting to expand this analysis by considering the severity levels of the disease, particularly using the Hoehn and Yahr (H&Y) scale [97] and the Unified Parkinson’s Disease Rating Scale (UPDRS) [98], to pool the detection performance of AI solutions.

The second limitation is the substantial heterogeneity and high inter-study variance observed. Although subgroup analyses explained part of this heterogeneity, it remained significant. It is necessary to explore other factors—such as processing techniques, the classifiers used, or the number and nature of the input data for the AI model—and to conduct multivariate meta-regression to further investigate the possible causes underlying this heterogeneity. It is also important to note that several comparisons could not be conducted or were performed with limited sample sizes (*n* < 10) due to a lack of data. Further studies are needed to consolidate the results presented.

The third limitation relates to the low level of evidence of the meta-analysis results. This is primarily due to the low representativeness of the populations included in the datasets and an incomplete validation process, resulting in a high level of risk across all included studies. It therefore appears important to follow the methodological recommendations provided by tools such as PROBAST+AI when conducting diagnostic test accuracy studies.

The fourth limitation concerns the writing task. Unlike the drawing tasks, which were defined separately (circle, meander, spiral, wave), handwriting was considered as a single task due to the small number of studies. However, some authors had explicitly distinguished between writing a letter, a word, a sentence, or a number. This distinction should be taken into account in future comparisons to refine the classification performance of PD patients based on handwriting.

Another limitation relates to the selection of the best-performing algorithm when multiple solutions were evaluated within the same study. Although this approach helps avoid non-independent observations, this choice may have introduced an optimistic bias. This bias, distinct from publication bias, could explain the asymmetry observed in the funnel plots regarding accuracy, specificity, and sensitivity. Consequently, pooled performance estimates should be interpreted with caution.

Our study focused solely on PD diagnosis based on handwriting and drawing tasks, excluding other symptoms such as tremors or speech and gait disorders. This limited the number of studies included.

Finally, it is worth mentioning the methodological limitations associated with the study selection process. The choice of inclusion and exclusion criteria and the search strategy (databases, keywords, selection procedure, etc.) may have resulted in the omission of some relevant studies.

## 5. Conclusions

This systematic review and meta-analysis highlights the high performance of AI methods in distinguishing PD patients from healthy subjects based on handwriting and drawing tasks. Additional analyses showed that several factors related to the data, tasks, and validation procedures were associated with the reported performance, while no systematic advantage was observed for any particular deep learning architecture. Despite these promising results, significant heterogeneity persisted for several performance metrics. Furthermore, the assessment of bias risk using PROBAST+AI and the evaluation of the certainty of evidence according to the GRADE approach revealed an overall very low level of confidence, requiring a cautious interpretation of the results and limiting, at this stage, their direct application to clinical practice. Nevertheless, this study identifies several methodological factors that may influence model performance and underscores the need for more standardized protocols, subject-wise data separation, and external validations on independent populations. These improvements should contribute to the development of more robust and generalizable AI models and help better determine the true potential of handwriting and drawing as non-invasive tools to aid in PD detection.

## Figures and Tables

**Table 1:** Keyword combination for each database.

Database	Keyword Combination
Google Scholar, IEEE Xplore, PubMed/MedLine, Science.gov	(Parkinson OR “parkinson’s disease” OR “Parkinson disease”) AND (“Artificial intelligence” OR “deep learning”) AND (handwriting OR spiral OR circle OR wave OR meander) AND (sensitivity OR specificity OR accuracy OR precision OR F1)
ScienceDirect	(Parkinson OR “parkinson’s disease”) AND “Artificial intelligence” AND handwriting AND (sensitivity OR specificity OR accuracy OR precision OR F1)

For Google Scholar, the “journal article” filter was used; for IEEE Xplore, the “Journal” filter was used; for ScienceDirect, the “Research articles” filter was used. No filters were applied for PubMed/MedLine and Science.gov.

**Table 2:** Detailed description of each included study.

Authors	Country	AI Method	Algorithm	Detailed Algorithm	Dataset	Subjects	Tasks	Validation
Abdullah et al. 2023 [60]	Iraq	DL+ML	CNN	TL (ResNet50 + VGG19 + InceptionV3) + GA + KNN	NewHandPD	264 (279 images for PD, 315 images for HC)	C, M, S	Hold-out (80% train/20% test)
Agrawal et al. 2024 [52]	India	DL+ML	CNN	VGG16 + SVM	NewHandPD	66 (31 PD/35 HC)	C, M, S	10-fold CV
Ahmed et al. 2026 [65]	Saudi Arabia	DL	CNN + RNN	CNN + BiLSTM	Spiral drawing PaHaW NewHandPD	77 (62 PD/15 HC) 75 (37 PD/38 HC) 66 (31 PD/35 HC)	C, M, H (l, w, s), S	Stratified 5-fold CV
Aldhyani et al. 2026 [66]	Saudi Arabia	DL	Transformer	ViT	Custom	63 (30 PD/33 HC)	E, H (n, w), S, W	Hold-out: (70% train/15% test/15% validation)
Ali et al. 2025 [63]	Pakistan	DL+ML	ANN	ChiGa-Net: VGG16 + χ^2^ feature refinement + GAONN	NewHandPD HandPD	66 (31 PD/35 HC) 92 (74 PD/18 HC)	M, S	Subject-wise LOSO CV
Allebawi et al. 2024 [23]	Tunisia	DL	RNN	BiLSTM	PaHaW	75 (37 PD/38 HC)	S, H (l, w, s)	10-fold CV
Allebawi et al. 2026 [61]	Iraq	DL	Siamese CNN	CNN + SN	Iraqi hand-drawing Spiral-Wave	60 (30 PD/30 HC) 55 (27 PD/28 HC)	E, H (num, l, w), S, W	Hold-out (70% train/30% test)
Bashir et al. 2026 [53]	India	DL	CNN	ResNet50 + MobileNetV2	NewHandPD	66 (31 PD/35 HC)	C, M, S	Hold-out (NA train/test)
El-Sayed et al. 2023 [50]	Egypt	DL	CNN + RNN	CNN + LSTM	NIATS NewHandPD	27 (15 PD/12 HC) 66 (31 PD/35 HC)	C, M, S, W	Hold-out (NA train/test)
Gazda et al. 2022 [67]	Slovakia	DL	CNN	CNN	NewHandPD PaHaW	66 (31 PD/35 HC) 69 (33 PD/36 HC)	M, S	Stratified subject-wise 5-fold CV
Gil-Martín et al. 2019 [68]	Spain	DL	CNN	CNN	Parkinson disease spiral drawing	77 (62 PD/15 HC)	S	Subject-wise 5-fold CV
Huang et al. 2024 [22]	Australia	DL	CNN	VGG19	NIATS	27 (15 PD/12 HC)	S, W	Hold-out (random 72 im. train/30 im. test)
Kamran et al. 2021 [17]	Pakistan	DL	CNN	AlexNet + VGG16 + ResNet50 + GoogLeNet	PaHaW HandPD NewHandPD Parkinson drawing	75 (37 PD/38 HC) 92 (74 PD/18 HC) 66 (31 PD/35 HC) 204 (102 PD/102 HC)	C, M, S, W	Hold-out (90% train/10% test)
Kansizoglou et al. 2025 [51]	Greece	DL	CNN	ResNet18	NewHandPD	66 (31 PD/35 HC)	C, M, S	5-fold CV
Kumar et al. 2026 [54]	India	DL	CNN	CNN	HandPD NewHandPD	NR (296 images for PD, 72 images for HC) NR (139 images for PD, 124 images for HC)	M, S	Hold-out (80% train/20% test)
Ma et al. 2022 [21]	China	DL	Transformer	Digital Ink Transformer	PaHaW	75 (37 PD/38 HC)	S	5-fold CV
Naz et al. 2023 [64]	Pakistan	DL+ML	CNN	CNN + SVM	HandPD NewHandPD Parkinson drawing	92 (74 PD/18 HC) 66 (31 PD/35 HC) 204 (102 PD/102 HC)	C, M, S, W	Hold-out (90% train/10% test)
Pereira et al. 2018 [16]	Brazil	DL	CNN	CNN	NewHandPD	34 (14 PD/20 HC)	C, M, S	Hold-out (50% train/50% test)
Saleh et al. 2025 [62]	Morocco	DL+ML	CNN	CNN + KNN	Spiral-Wave	NA (102 images for PD, 102 images for HC)	S, W	Hold-out (70% train/30% test)
Saravanan et al. 2023 [55]	India	DL	CNN	VGG19 + Inception	Custom	367 (175 PD/192 HC)	S, W	Hold-out (70% train/30% test)
Shanthappa et al. 2025 [56]	India	DL	CNN	InceptionV3	Spiral drawing	NA (160 spiral for PD and HC)	S	Hold-out (NA train/test)
Shermy et al. 2025 [57]	India	DL	CNN + Transformer + GNN	GLCST	NewHandPD PaHaW HandPD	NA (315 image for PD, 279 images for HC) 75 (37 PD/38 HC) 92 (74 PD/18 HC)	C, H (l, w, s), M, S	5-fold CV
Shyamala et al. 2025 [58]	India	DL	CNN	Hybrid CNN + TL	Parkinson drawing	55 (27 PD/28 HC)	S, W	Hold-out: (70% train/20% test/10% validation)
Varalakshmi et al. 2022 [59]	India	DL+ML	CNN	ResNet50 + SVM	Parkinson drawing	55 (27 PD/28 HC)	S	Hold-out (70% train/30% test)
Wang et al. 2023 [45]	China	DL	Transformer	CAS-Transformer	HandPD	92 (74 PD/18 HC)	M, S	Hold-out (70% train/30% test)
Wang et al. 2024 [44]	Belgium	DL	CNN + RNN	LSTM + CNN	DraWritePD PaHaW	49 (20 PD/29 HC) 75 (37 PD/38 HC)	M, S	5-fold CV
Yu et al. 2026 [46]	China	DL	CNN + Transformer	BDINet: CoorLGNet + SIFT + MFIM	HandPD NewHandPD Short Text	92 (74 PD/18 HC) 66 (31 PD/35 HC) 154 (89 PD/65 HC)	C, H, S, W	Hold-out (70% train/30% test)
Zhao et al. 2023 [47]	China	DL	CNN + RNN	SENN	NewHandPD	66 (31 PD/35 HC)	C, M, S	Hold-out (80% train/20% test)
Zhao et al. 2026 [48]	China	DL	CNN	HierFuse: temporal and spatial CNN + HGF	NewHandPD	66 (31 PD/35 HC)	C, M, S	5-fold CV
Zhu et al. 2025 [49]	China	DL	CNN + RNN	CNN + RNN	Custom NewHandPD	58 (28 PD/30 HC) 66 (31 PD/35 HC)	S, W	Stratified subject-wise 10-fold CV

C: circle; W: wave; E: ellipse; M: meander; S: spiral; H: handwriting; l: letter; w: word; s: sentence; num: number; DL: deep learning; ML: machine learning; PD: Parkinson’s disease; HC: healthy control; BE: beta-elliptical; im: image; CV: cross validation; NA: not available; RF: random forest; LOSO: leave one subject out; GA: genetic algorithm; KNN: k-nearest neighbors; ViT: Vision Transformer; SN: Siamese Network; TL: Transfer learning; SVM: support vector machine; SENN: Self-explaining neural network; LSTM: long short-term memory; BiLSTM: Bidirectional long short-term memory; GLCST: Graph Learning-based Convolutional Sparse Transformer; BDINet: Bidirectional dynamic interaction network; CNN: convolutional neural network; VGG: visual geometry group; RNN: recurrent neural network; HGF: hierarchical gated fusion; SIFT: Scale-Invariant Feature Transform; MFIM: Multi-angle Feature Interaction Module; SN: Siamese network; GAONN: Genetic algorithm-optimized neural network; ANN: artificial neural network.

**Table 3:** Performance parameters used by each included study.

Authors	Nb Algorithms	Accuracy	Specificity	Sensitivity	Precision	F1-Score
Abdullah et al. 2023 [60]	1	X		X	X	
Agrawal et al. 2024 [52]	4	X	X	X		
Ahmed et al. 2026 [65]	3	X		X	X	X
Aldhyani et al. 2026 [66]	5	X		X	X	X
Ali et al. 2025 [63]	4	X	X	X		
Allebawi et al. 2024 [23]	4	X				
Allebawi et al. 2026 [61]	7	X		X	X	X
Bashir et al. 2026 [53]	1	X		X	X	X
El-Sayed et al. 2023 [50]	5	X				
Gazda et al. 2022 [67]	9	X				
Gil-Martín et al. 2019 [68]	1	X				X
Huang et al. 2024 [22]	2	X		X	X	
Kamran et al. 2021 [17]	14	X				
Kansizoglou et al. 2025 [51]	4	X	X	X	X	
Kumar et al. 2026 [54]	9	X	X	X		
Ma et al. 2022 [21]	1	X				
Naz et al. 2023 [64]	1	X	X	X	X	X
Pereira et al. 2018 [16]	5	X	X	X		
Saleh et al. 2025 [62]	3	X		X	X	X
Saravanan et al. 2023 [55]	1	X				
Shanthappa et al. 2025 [56]	1	X				
Shermy et al. 2025 [57]	3	X	X	X	X	X
Shyamala et al. 2025 [58]	1	X				
Varalakshmi et al. 2022 [59]	1	X	X	X	X	X
Wang et al. 2023 [45]	2	X	X	X	X	X
Wang et al. 2024 [44]	3	X	X	X		
Yu et al. 2026 [46]	3	X		X	X	X
Zhao et al. 2023 [47]	3	X	X	X		
Zhao et al. 2026 [48]	3	X		X	X	X
Zhu et al. 2025 [49]	4	X	X	X		X

Nb: number. X indicates that authors reported a value for this performance metric.

**Table 4:** Results of the meta-analysis by performance parameter.

Parameter	Pooled	95% CI	τ^2^	I^2^	*n*
Accuracy	93.17%	91.78–94.33%	0.77	84.81%	108
Specificity	91.62%	88.34–94.03%	0.95	75.32%	42
Sensitivity	93.93%	92.42–95.15%	0.49	56.31%	71
Precision	95.76%	94.00–97.02%	0.41	40.74%	36
F1-score	95.02%	92.78–96.58%	0.68	57.93%	36

95% CI: 95% confidence interval; *n*: number of available data.

**Table 5:** AI method (DL vs. hybrid DL+ML) comparison of pooled values and heterogeneity parameters obtained for each performance metric.

Parameter	AI Method	Pooled	95% CI	τ^2^	I^2^	*n*	z	*p*(Wald)
Accuracy	DL	92.74%	91.20–94.03%	0.730	84.59%	94	1.580	0.114
	Hybrid	95.59%	91.99–97.62%	1.075	84.73%	14		
Specificity	DL	90.04%	85.97–93.02%	0.815	72.58%	32	1.912	0.056
	Hybrid	96.01%	90.35–98.41%	1.453	79.65%	10		
Sensitivity	DL	93.70%	92.05–95.03%	0.360	46.81%	57	0.345	0.730
	Hybrid	94.52%	90.35–96.94%	0.897	75.04%	14		
Precision	DL	95.28%	93.35–96.67%	0.248	29.56%	30	1.323	0.186
	Hybrid	97.35%	92.19–99.13%	1.025	56.41%	6		
F1-score	DL	94.45%	91.94–96.22%	0.542	50.30%	31	1.276	0.202
	Hybrid	97.02%	90.42–99.12%	1.241	68.74%	5		

95% CI: 95% confidence interval; *n*: number of available data.

**Table 6:** Algorithm comparison of pooled values and heterogeneity parameters obtained for each performance metric.

Parameter	Algorithm	Pooled	95% CI	τ^2^	I^2^	*n*
Accuracy	CNN	92.04%	89.82–93.81%	0.663	87.24%	48
	Combined CNN	93.71%	91.25–95.51%	0.620	71.82%	31
	Transformer	96.29%	93.38–97.95%	0.000	0.00%	8
Specificity	CNN	93.18%	89.78–95.50%	0.411	57.29%	16
	Combined CNN	91.86%	85.78–95.48%	0.790	68.49%	13
	Transformer	89.93%	79.75–95.30%	0.000	0.00%	2
Sensitivity	CNN	95.05%	93.01–96.52%	0.406	53.53%	24
	Combined CNN	94.37%	91.65–96.24%	0.489	46.26%	26
	Transformer	90.08%	84.19–93.94%	0.000	0.00%	7
Precision	CNN	93.48%	89.76–95.91%	0.141	30.79%	8
	Combined CNN	96.99%	94.88–98.24%	0.264	22.18%	16
	Transformer	94.44%	88.95–97.29%	0.000	0.00%	7
F1-score	CNN	92.82%	86.73–96.24%	0.284	48.05%	5
	Combined CNN	95.14%	91.60–97.23%	0.830	58.13%	19
	Transformer	95.11%	90.94–97.41%	0.000	0.00%	7

95% CI: 95% confidence interval; *n*: number of available data.

**Table 7:** Dataset comparison of pooled values and heterogeneity parameters obtained for each performance metric.

Parameter	Dataset	Pooled	95% CI	τ^2^	I^2^	*n*
Accuracy	Combined	95.03%	92.43–96.76%	0.146	49.67%	6
	HandPD	92.13%	88.90–94.48%	0.208	54.15%	11
	NewHandPD	93.51%	91.25–95.22%	0.783	82.40%	40
	PaHaW	88.09%	81.11–92.73%	0.991	94.69%	16
	Parkinson’s Drawing	96.59%	90.03–98.88%	1.206	80.77%	5
Specificity	Combined	95.40%	91.61–97.52%	0.000	0.00%	4
	HandPD	83.88%	76.76–89.13%	0.000	0.00%	7
	NewHandPD	91.15%	85.54–94.73%	1.339	82.41%	23
	PaHaW	93.85%	65.14–99.20%	1.514	59.68%	2
Sensitivity	Combined	95.86%	93.34–97.45%	0.000	0.00%	4
	HandPD	93.99%	89.63–96.58%	0.451	67.21%	8
	NewHandPD	93.48%	90.87–95.38%	0.577	64.81%	30
	PaHaW	96.19%	89.85–98.63%	0.000	0.00%	3
Precision	HandPD	97.97%	93.32–99.41%	0.602	49.48%	3
	NewHandPD	95.65%	91.81–97.73%	0.564	57.71%	9
	PaHaW	97.94%	90.11–99.60%	0.000	0.00%	2
F1-score	HandPD	97.36%	94.04–98.86%	0.183	31.46%	3
	NewHandPD	94.49%	88.78–97.38%	1.003	76.44%	9
	PaHaW	97.87%	90.23–99.56%	0.000	0.00%	2

95% CI: 95% confidence interval; *n*: number of available data.

**Table 8:** Drawing and handwriting task comparison of pooled values and heterogeneity parameters obtained for each performance metric.

Parameter	Task	Pooled	95% CI	τ^2^	I^2^	*n*
Accuracy	Circle	84.89%	75.92–90.92%	0.365	64.73%	7
	Combined	96.23%	94.87–97.23%	0.475	65.76%	32
	Handwriting	88.89%	82.13–93.31%	0.952	93.76%	17
	Meander	92.40%	89.67–94.45%	0.269	66.81%	15
	Spiral	92.97%	90.18–95.01%	0.685	80.35%	29
	Wave	87.90%	82.09–92.00%	0.062	18.10%	6
Specificity	Circle	76.00%	55.45–88.96%	0.721	64.91%	5
	Combined	95.61%	91.74–97.71%	0.649	57.05%	11
	Meander	89.82%	83.96–93.71%	0.449	64.31%	11
	Spiral	92.37%	85.49–96.13%	1.199	81.23%	13
Sensitivity	Circle	87.11%	80.56–91.68%	0.000	0.00%	6
	Combined	95.97%	93.72–97.44%	0.620	67.97%	18
	Handwriting	94.44%	87.37–97.66%	0.000	0.00%	8
	Meander	93.85%	90.90–95.89%	0.174	34.29%	12
	Spiral	93.09%	89.30–95.60%	0.653	65.15%	20
	Wave	89.71%	80.54–94.84%	0.000	0.00%	5
Precision	Combined	97.24%	95.65–98.25%	0.189	31.85%	12
	Handwriting	94.99%	88.10–97.98%	0.000	0.00%	8
	Spiral	94.76%	88.41–97.72%	0.817	57.85%	8
	Wave	92.57%	80.62–97.39%	0.000	0.00%	4
F1-score	Combined	97.54%	96.17–98.43%	0.115	20.13%	11
	Handwriting	94.25%	87.03–97.57%	0.000	0.00%	8
	Spiral	92.99%	84.98–96.89%	1.136	77.02%	9
	Wave	89.01%	79.10–94.55%	0.000	0.00%	4

95% CI: 95% confidence interval; *n*: number of available data.

**Table 9:** Validation method comparison of pooled values and heterogeneity parameters obtained for each performance metric.

Parameter	Validation	Pooled	95% CI	τ^2^	I^2^	*n*	z	*p*(Wald)
Accuracy	Cross-validation	95.85%	94.06–97.12%	0.492	70.67%	21	−2.107	0.035 *
	Hold out	93.40%	91.70–94.77%	0.630	74.21%	66		
Specificity	Cross-validation	95.63%	92.07–97.64%	0.687	67.03%	12	−2.245	0.025 *
	Hold out	89.57%	83.72–93.48%	1.039	77.81%	22		
Sensitivity	Cross-validation	96.50%	94.17–97.92%	0.633	57.10%	16	−2.294	0.022 *
	Hold out	93.27%	91.17–94.90%	0.412	52.53%	44		
Precision	Cross-validation	95.42%	90.10–97.95%	0.793	72.24%	7	0.232	0.816
	Hold out	95.77%	93.51–97.26%	0.351	29.83%	26		
F1-score	Cross-validation	95.60%	88.59–98.38%	1.099	72.09%	6	0.129	0.897
	Hold out	95.76%	93.31–97.34%	0.469	42.92%	23		

95% CI: 95% confidence interval; *n*: number of available data. * indicates a significant difference (*p* < 0.05).

**Table 10:** Sensitivity analysis—one-by-one exclusion of the proposed algorithms.

Parameter	Algorithm Excluded—Dataset—Task	Pooled Performance		I^2^	
		LOO	All	LOO	All
Accuracy	Shyamala et al. [58]—Parkinson’s Drawing—Combined S+W	92.98%	93.17%	84.02%	84.81%
Specificity	Shermy et al. [57]—NewHandPD—Combined C+M+S	91.08%	91.62%	73.06%	75.32%
Sensitivity	Varalakshmi et al. [59]—Parkinson drawing—S	93.61%	93.93%	51.16%	56.31%
Precision	Varalakshmi et al. [59]—Parkinson drawing—S	95.31%	95.76%	28.19%	40.74%
F1-score	Varalakshmi et al. [59]—Parkinson drawing—S	94.50%	95.02%	48.47%	57.93%

C: circle; M: meander; S: spiral; W: wave; LOO: leave-one-out.

**Table 11:** Sensitivity analysis—one-by-one exclusion of included studies (all proposed algorithms).

Parameter	Study Excluded	Pooled Performance		I^2^	
		LOSO	All	LOSO	All
Accuracy	Gazda et al. 2022 [67]	93.95%	93.17%	74.75%	84.81%
Specificity	Pereira et al. 2018 [16]	93.07%	91.62%	65.07%	75.32%
Sensitivity	Pereira et al. 2018 [16]	94.40%	93.93%	53.06%	56.31%
Precision	Zhao et al. 2026 [48]	96.38%	95.76%	30.23%	40.74%
F1-score	Zhu et al. 2025 [49]	95.90%	95.02%	46.87%	57.93%

LOSO: leave-one-study-out.

**Table 12:** Detailed GRADE analysis.

Performance Parameter	Number of Studies	Certainty Assessment						Effect			Overall Level of Evidence
		Study Design	Publication Bias (Egger’s Test)	Indirectness ^a^	Inconsistency ^b^	Imprecision ^c^	Risk of Bias ^d^	*n*	Event Rate	(95% CI)	
Accuracy	30	DTAS	Serious (*p* < 0.001)	Serious	Serious (I^2^ = 84.81%)	Not serious	Serious	108	93.17%	91.78–94.33%	Very low ⬤◯◯◯
Specificity	12	DTAS	Serious (*p* < 0.001)	Serious	Serious (I^2^ = 75.32%)	Not serious	Serious	42	91.62%	88.34–94.03%	Very low ⬤◯◯◯
Sensitivity	21	DTAS	Serious (*p* = 0.023)	Serious	Serious (I^2^ = 56.31%)	Not serious	Serious	71	93.93%	92.42–95.15%	Very low ⬤◯◯◯
Precision	14	DTAS	Not serious (*p* = 0.836)	Serious	Not serious (I^2^ = 40.74%)	Not serious	Serious	36	95.76%	94.00–97.02%	Low ⬤⬤◯◯
F1 score	13	DTAS	Not serious (*p* = 0.613)	Serious	Serious (I^2^ = 57.93%)	Not serious	Serious	36	95.02%	92.78–96.58%	Very low ⬤◯◯◯

DTAS: diagnostic test accuracy study; *n*: number of available data; 95% CI: 95% confidence interval. GRADE overall quality significance: ⬤◯◯◯ = Very low; ⬤⬤◯◯ = Low; ⬤⬤⬤◯ = Moderate; ⬤⬤⬤⬤ = High. ^a^ Studied population correspond to the population in study. ^b^ Serious if I^2^ > 50%. ^c^ Serious if 95% CI Range Difference > 50% of Event Rate. ^d^ Studies have at least a fair critical appraisal score.

## Data Availability

The original contributions presented in this study are included in the article. Further inquiries can be directed to the corresponding author.

## Supplementary Materials

The following supporting information can be downloaded at: https://www.mdpi.com/article/10.3390/s26216893/s1, the five Supplementary Files (Figures S1–S5) attached to this article contain detailed forest plots of each performance parameter. Figure S1: Forest plot of logit transformed accuracy pooled across studies. Each square represents the logit transformed accuracy of one algorithm. The horizontal bars represent the 95% confidence interval. The diamond at the bottom represents the pooled accuracy. References [16,17,21,22,23,44,45,46,47,48,49,50,51,52,53,54,55,56,57,58,59,60,61,62,63,64,65,66,67,68]; Figure S2: Forest plot of logit transformed specificity pooled across studies. Each square represents the logit transformed specificity of one algorithm. The horizontal bars represent the 95% confidence interval. The diamond at the bottom represents the pooled specificity. References [16,44,45,47,48,49,51,52,54,57,59,63,64]; Figure S3: Forest plot of logit transformed sensitivity pooled across studies. Each square represents the logit transformed sensitivity of one algorithm. The horizontal bars represent the 95% confidence interval. The diamond at the bottom represents the pooled sensitivity. References [16,44,45,46,47,48,49,51,52,53,54,57,59,60,61,62,63,64,65,66]; Figure S4: Forest plot of logit transformed precision pooled across studies. Each square represents the logit transformed precision of one algorithm. The horizontal bars represent the 95% confidence interval. The diamond at the bottom represents the pooled precision. References [22,45,46,48,51,53,57,60,61,62,65,66]; Figure S5: Forest plot of logit transformed F1-score pooled across studies. Each square represents the logit transformed F1-score of one algorithm. The horizontal bars represent the 95% confidence interval. The diamond at the bottom represents the pooled F1-score. References [45,46,48,49,53,57,59,61,62,64,65,66,68].

## Abbreviations

The following abbreviations are used in this manuscript:

AI	Artificial Intelligence
ANN	Artificial Neural Network
BDINet	Bidirectional Dynamic Interaction Network
BE	Beta-Elliptical
BiLSTM	Bidirectional Long Short-Term Memory
C	Circle
CI	Confidence Interval
CNN	Convolutional Neural Networks
CV	Cross-Validation
DL	Deep Learning
DTAS	Diagnostic Test Accuracy Study
E	Ellipse
FN	False Negatives
FP	False Positives
GA	Genetic Algorithm
GAONN	Genetic Algorithm-Optimized Neural Network
GLCST	Graph Learning-Based Convolutional Sparse Transformer
GRADE	Grading Of Recommendations Assessment, Development, And Evaluation
H	Handwriting
HC	Healthy Controls
HGF	Hierarchical Gated Fusion
im	Image
KNN	K-Nearest Neighbors
l	Letter
LOO	Leave One Out
LOSO	Leave-One-Subject-Out
LSTM	Long Short-Term Memory
M	Meander
MFIM	Multi-Angle Feature Interaction Module
ML	Machine Learning
num	Number
NA	Not Available
Nb	Number
PD	Parkinson’s Disease
PRISMA	Preferred Reporting Items For Systematic Reviews And Meta-Analyses
PROBAST+AI	Prediction Model Study Risk Of Bias Assessment Tool + Artificial Intelligence
REML	Restricted Maximum Likelihood
RF	Random Forest
RNN	Recurrent Neural Network
S	Spiral
s	Sentence
SE	Standard Error
SENN	Self-Explaining Neural Network
SIFT	Scale-Invariant Feature Transform
SN	Siamese Network
SVM	Support Vector Machine
TL	Transfer Learning
TN	True Negatives
TP	True Positives
VGG	Visual Geometry Group
ViT	Vision Transformer
W	Wave
w	Word

## Appendix A

The following table displays detailed results of pairwise mixed-effects meta-regression for different DL architectures.

**Table A1:** Algorithm architecture comparison of pooled values and heterogeneity parameters obtained for the five performance parameters.

Parameter	Comparison	β ^a^ (95% CI)	z	*p*(Wald)	QM	*p*(QM)
Accuracy	Combined CNN vs. CNN	0.258 (−0.192 to 0.709)	1.125	0.261	1.265	0.261
	Transformer vs. CNN	0.866 (−0.090 to 1.821)	1.776	0.076	3.155	0.076
	Transformer vs. Combined CNN	0.621 (−0.333 to 1.575)	1.276	0.202	1.628	0.202
Specificity	Combined CNN vs. CNN	−0.250 (−1.005 to 0.504)	−0.65	0.516	0.423	0.516
	Transformer vs. CNN	−0.431 (−1.679 to 0.816)	−0.677	0.498	0.459	0.498
	Transformer vs. Combined CNN	−0.242 (−1.788 to 1.303)	−0.307	0.759	0.094	0.759
Sensitivity	Combined CNN vs. CNN	−0.145 (−0.706 to 0.415)	−0.507	0.612	0.257	0.612
	Transformer vs. CNN	−0.720 (−1.605 to 0.166)	−1.593	0.111	2.539	0.111
	Transformer vs. Combined CNN	−0.586 (−1.531 to 0.360)	−1.214	0.225	1.474	0.225
Precision	Combined CNN vs. CNN	0.820 (0.075 to 1.565)	2.157	0.031 *	4.653	0.031
	Transformer vs. CNN	0.136 (−0.796 to 1.067)	0.285	0.775	0.081	0.775
	Transformer vs. Combined CNN	−0.700 (−1.689 to 0.290)	−1.386	0.166	1.921	0.166
F1-score	Combined CNN vs. CNN	0.367 (−0.687 to 1.422)	0.683	0.495	0.467	0.495
	Transformer vs. CNN	0.372 (−0.620 to 1.364)	0.735	0.463	0.540	0.463
	Transformer vs. Combined CNN	−0.158 (−1.323 to 1.007)	−0.265	0.791	0.070	0.791

^a^: The β values were reported on a logit scale. 95% CI: 95% confidence interval. * indicates a significant difference.

## Appendix B

The following table displays detailed results of pairwise mixed-effects meta-regression for the different datasets.

**Table A2:** Datasets comparison of pooled values and heterogeneity parameters obtained for the five performance parameters.

Parameter	Comparison	β ^a^ (95% CI)	z	*p*(Wald)	QM	*p*(QM)
Accuracy	Custom vs. Combined	−0.494 (−1.210 to 0.221)	−1.353	0.176	1.832	0.176
	HandPD vs. Combined	−0.513 (−1.105 to 0.078)	−1.701	0.089	2.894	0.089
	NewHandPD vs. Combined	−0.411 (−1.241 to 0.418)	−0.972	0.331	0.945	0.331
	PaHaW vs. Combined	−1.115 (−2.043 to −0.186)	−2.353	0.019 *	5.539	0.019
	Parkinson’s Drawing vs. Combined	0.480 (−0.595 to 1.554)	0.875	0.381	0.766	0.381
	HandPD vs. Custom	−0.028 (−0.721 to 0.665)	−0.079	0.937	0.006	0.937
	NewHandPD vs. Custom	0.024 (−0.844 to 0.893)	0.055	0.956	0.003	0.956
	PaHaW vs. Custom	−0.680 (−1.647 to 0.287)	−1.378	0.168	1.9	0.168
	Parkinson’s Drawing vs. Custom	0.864 (−0.314 to 2.043)	1.438	0.151	2.067	0.151
	NewHandPD vs. HandPD	0.105 (−0.543 to 0.753)	0.317	0.751	0.1	0.751
	PaHaW vs. HandPD	−0.603 (−1.351 to 0.146)	−1.578	0.115	2.491	0.115
	Parkinson’s Drawing vs. HandPD	0.997 (0.019 to 1.974)	1.998	0.046 *	3.993	0.046
	PaHaW vs. NewHandPD	−0.697 (−1.304 to −0.091)	−2.254	0.024 *	5.08	0.024
	Parkinson’s Drawing vs. NewHandPD	0.746 (−0.296 to 1.789)	1.404	0.16	1.971	0.16
	Parkinson’s Drawing vs. PaHaW	1.364 (0.152 to 2.576)	2.206	0.027 *	4.865	0.027
Specificity	Custom vs. Combined	−1.021 (−1.930 to −0.113)	−2.203	0.028 *	4.854	0.028
	HandPD vs. Combined	−1.382 (−2.167 to −0.597)	−3.45	<0.001	11.901	<0.001
	NewHandPD vs. Combined	−0.896 (−2.302 to 0.511)	−1.248	0.212	1.557	0.212
	PaHaW vs. Combined	−0.801 (−1.988 to 0.386)	−1.323	0.186	1.75	0.186
	Parkinson’s Drawing vs. Combined	0.861 (−0.008 to 1.729)	1.943	0.052	3.774	0.052
	HandPD vs. Custom	−0.361 (−1.149 to 0.428)	−0.896	0.37	0.803	0.37
	NewHandPD vs. Custom	0.285 (−1.198 to 1.769)	0.377	0.706	0.142	0.706
	PaHaW vs. Custom	0.201 (−0.909 to 1.311)	0.355	0.722	0.126	0.722
	Parkinson’s Drawing vs. Custom	1.882 (1.011 to 2.754)	4.233	<0.001	17.914	<0.001
	NewHandPD vs. HandPD	0.459 (−0.626 to 1.544)	0.828	0.407	0.686	0.407
	PaHaW vs. HandPD	0.562 (−0.449 to 1.573)	1.089	0.276	1.186	0.276
	Parkinson’s Drawing vs. HandPD	2.243 (1.501 to 2.984)	5.925	<0.001	35.106	<0.001
	PaHaW vs. NewHandPD	0.367 (−1.721 to 2.454)	0.344	0.731	0.119	0.731
	Parkinson’s Drawing vs. NewHandPD	1.559 (−0.848 to 3.966)	1.269	0.204	1.611	0.204
	Parkinson’s Drawing vs. PaHaW	1.167 (−2.084 to 4.418)	0.704	0.482	0.495	0.482
Sensitivity	Custom vs. Combined	−1.016 (−1.807 to −0.225)	−2.518	0.012 *	6.342	0.012
	HandPD vs. Combined	−0.539 (−1.478 to 0.400)	−1.125	0.261	1.265	0.261
	NewHandPD vs. Combined	−0.626 (−1.587 to 0.335)	−1.277	0.201	1.632	0.201
	PaHaW vs. Combined	0.089 (−1.074 to 1.251)	0.149	0.881	0.022	0.881
	Parkinson’s Drawing vs. Combined	1.454 (0.489 to 2.419)	2.952	0.003 *	8.717	0.003
	HandPD vs. Custom	0.551 (−0.356 to 1.458)	1.191	0.234	1.418	0.234
	NewHandPD vs. Custom	0.495 (−0.416 to 1.405)	1.065	0.287	1.135	0.287
	PaHaW vs. Custom	1.105 (−0.110 to 2.319)	1.782	0.075	3.176	0.075
	Parkinson’s Drawing vs. Custom	2.470 (1.443 to 3.497)	4.712	<0.001	22.207	<0.001
	NewHandPD vs. HandPD	−0.111 (−0.838 to 0.616)	−0.298	0.765	0.089	0.765
	PaHaW vs. HandPD	0.580 (−0.846 to 2.007)	0.797	0.425	0.636	0.425
	Parkinson’s Drawing vs. HandPD	1.846 (0.184 to 3.508)	2.177	0.03 *	4.738	0.03
	PaHaW vs. NewHandPD	0.680 (−0.757 to 2.117)	0.927	0.354	0.859	0.354
	Parkinson’s Drawing vs. NewHandPD	1.932 (0.192 to 3.673)	2.176	0.03 *	4.733	0.03
	Parkinson’s Drawing vs. PaHaW	1.365 (0.030 to 2.700)	2.005	0.045 *	4.019	0.045
Precision	Custom vs. Combined	−3.086 (−6.157 to −0.015)	−1.97	0.049 *	3.88	0.049
	HandPD vs. Combined	−1.404 (−4.806 to 1.998)	−0.809	0.419	0.654	0.419
	NewHandPD vs. Combined	−2.192 (−5.409 to 1.024)	−1.336	0.182	1.785	0.182
	PaHaW vs. Combined	−1.420 (−4.653 to 1.814)	−0.86	0.39	0.74	0.39
	NewHandPD vs. Custom	0.883 (−0.683 to 2.449)	1.105	0.269	1.221	0.269
	PaHaW vs. Custom	1.666 (−0.441 to 3.774)	1.55	0.121	2.401	0.121
	Parkinson’s Drawing vs. Custom	2.398 (0.853 to 3.943)	3.041	0.002 *	9.249	0.002
	NewHandPD vs. HandPD	−0.782 (−2.179 to 0.616)	−1.096	0.273	1.202	0.273
	PaHaW vs. HandPD	0.048 (−2.168 to 2.265)	0.043	0.966	0.002	0.966
	Parkinson’s Drawing vs. HandPD	0.716 (−1.413 to 2.845)	0.659	0.51	0.435	0.51
	PaHaW vs. NewHandPD	0.806 (−1.259 to 2.872)	0.765	0.444	0.585	0.444
	Parkinson’s Drawing vs. NewHandPD	1.504 (−0.313 to 3.322)	1.623	0.105	2.633	0.105
	Parkinson’s Drawing vs. PaHaW	0.731 (−1.117 to 2.580)	0.776	0.438	0.602	0.438
F1-score	Custom vs. Combined	−2.712 (−4.904 to −0.520)	−2.425	0.015 *	5.879	0.015
	HandPD vs. Combined	−1.092 (−3.505 to 1.321)	−0.887	0.375	0.787	0.375
	NewHandPD vs. Combined	−1.859 (−4.834 to 1.115)	−1.225	0.221	1.501	0.221
	PaHaW vs. Combined	−0.875 (−3.515 to 1.765)	−0.65	0.516	0.422	0.516
	HandPD vs. Custom	1.539 (0.618 to 2.461)	3.274	0.001 *	10.717	0.001
	NewHandPD vs. Custom	0.761 (−0.440 to 1.961)	1.242	0.214	1.543	0.214
	PaHaW vs. Custom	1.837 (0.110 to 3.564)	2.084	0.037 *	4.345	0.037
	Parkinson’s Drawing vs. Custom	2.605 (1.739 to 3.472)	5.894	<0.001	34.737	<0.001
	NewHandPD vs. HandPD	−0.873 (−2.351 to 0.605)	−1.158	0.247	1.34	0.247
	PaHaW vs. HandPD	0.244 (−1.625 to 2.113)	0.256	0.798	0.065	0.798
	Parkinson’s Drawing vs. HandPD	0.986 (−0.343 to 2.315)	1.454	0.146	2.113	0.146
	PaHaW vs. NewHandPD	1.037 (−1.213 to 3.287)	0.903	0.366	0.816	0.366
	Parkinson’s Drawing vs. NewHandPD	1.753 (−0.437 to 3.943)	1.569	0.117	2.462	0.117
	Parkinson’s Drawing vs. PaHaW	0.769 (−0.938 to 2.475)	0.883	0.377	0.779	0.377

^a^: The β values were reported on a logit scale. 95% CI: 95% confidence interval. * indicates a significant difference.

## Appendix C

The following table displays detailed results of pairwise mixed-effects meta-regression for the different drawing and handwriting tasks.

**Table A3:** Tasks comparison of pooled values and heterogeneity parameters obtained for the five performance parameters.

Parameters	Tasks	β ^a^ (95% CI)	z	*p*(Wald)	QM	*p*(QM)
Accuracy	Combined vs. Circle	1.499 (0.804 to 2.194)	4.226	<0.001 *	17.856	<0.001
	Handwriting vs. Circle	0.265 (−0.640 to 1.171)	0.574	0.566	0.33	0.566
	Meander vs. Circle	0.788 (0.149 to 1.426)	2.419	0.016 *	5.85	0.016
	Spiral vs. Circle	0.818 (0.039 to 1.598)	2.057	0.040 *	4.231	0.040
	Wave vs. Circle	0.419 (−0.397 to 1.234)	1.006	0.314	1.013	0.314
	Handwriting vs. Combined	−1.254 (−1.849 to −0.658)	−4.129	<0.001 *	17.046	<0.001
	Meander vs. Combined	−0.721 (−1.201 to −0.241)	−2.947	0.003 *	8.682	0.003
	Spiral vs. Combined	−0.676 (−1.162 to −0.189)	−2.72	0.007 *	7.398	0.007
	Wave vs. Combined	−1.052 (−1.842 to −0.263)	−2.614	0.009 *	6.833	0.009
	Meander vs. Handwriting	0.567 (−0.052 to 1.186)	1.796	0.072	3.226	0.072
	Spiral vs. Handwriting	0.553 (−0.085 to 1.191)	1.7	0.089	2.891	0.089
	Wave vs. Handwriting	0.222 (−0.793 to 1.237)	0.429	0.668	0.184	0.668
	Spiral vs. Meander	0.043 (−0.491 to 0.577)	0.157	0.876	0.025	0.876
	Wave vs. Meander	−0.376 (−1.077 to 0.324)	−1.053	0.292	1.108	0.292
	Wave vs. Spiral	−0.346 (−1.239 to 0.546)	−0.761	0.447	0.579	0.447
Specificity	Combined vs. Circle	1.932 (0.792 to 3.072)	3.322	<0.001 *	11.037	<0.001
	Spiral vs. Circle	1.325 (0.044 to 2.606)	2.028	0.043 *	4.111	0.043
	Wave vs. Circle	0.809 (−1.388 to 3.006)	0.722	0.471	0.521	0.471
	Spiral vs. Combined	−0.656 (−1.682 to 0.371)	−1.252	0.211	1.566	0.211
	Wave vs. Combined	−1.119 (−3.151 to 0.914)	−1.079	0.281	1.164	0.281
	Wave vs. Spiral	−0.532 (−3.044 to 1.979)	−0.415	0.678	0.172	0.678
Sensitivity	Combined vs. Circle	1.156 (0.270 to 2.041)	2.557	0.011 *	6.536	0.011
	Handwriting vs. Circle	0.922 (−0.101 to 1.946)	1.766	0.077	3.12	0.077
	Meander vs. Circle	0.747 (0.056 to 1.437)	2.12	0.034 *	4.496	0.034
	Spiral vs. Circle	0.580 (−0.321 to 1.481)	1.262	0.207	1.593	0.207
	Wave vs. Circle	0.255 (−0.636 to 1.146)	0.561	0.575	0.314	0.575
	Handwriting vs. Combined	−0.289 (−1.441 to 0.863)	−0.491	0.623	0.241	0.623
	Meander vs. Combined	−0.350 (−1.036 to 0.337)	−0.999	0.318	0.998	0.318
	Spiral vs. Combined	−0.573 (−1.242 to 0.096)	−1.679	0.093	2.82	0.093
	Wave vs. Combined	−0.921 (−2.040 to 0.198)	−1.613	0.107	2.601	0.107
	Meander vs. Handwriting	−0.133 (−1.164 to 0.898)	−0.253	0.801	0.064	0.801
	Spiral vs. Handwriting	−0.285 (−1.448 to 0.878)	−0.48	0.631	0.23	0.631
	Wave vs. Handwriting	−0.667 (−1.835 to 0.500)	−1.12	0.263	1.255	0.263
	Spiral vs. Meander	−0.228 (−0.926 to 0.470)	−0.64	0.522	0.41	0.522
	Wave vs. Meander	−0.505 (−1.423 to 0.413)	−1.079	0.281	1.164	0.281
	Wave vs. Spiral	−0.346 (−1.480 to 0.787)	−0.599	0.549	0.359	0.549
Precision	Handwriting vs. Combined	−0.591 (−1.676 to 0.493)	−1.069	0.285	1.142	0.285
	Spiral vs. Combined	−0.686 (−1.620 to 0.247)	−1.441	0.150	2.076	0.150
	Wave vs. Combined	−1.008 (−2.265 to 0.249)	−1.571	0.116	2.468	0.116
	Spiral vs. Handwriting	−0.048 (−1.408 to 1.312)	−0.069	0.945	0.005	0.945
	Wave vs. Handwriting	−0.419 (−1.863 to 1.025)	−0.569	0.569	0.324	0.569
	Wave vs. Spiral	−0.362 (−1.953 to 1.229)	−0.446	0.656	0.199	0.656
F1-score	Handwriting vs. Combined	−0.865 (−1.892 to 0.163)	−1.65	0.099	2.722	0.099
	Spiral vs. Combined	−1.157 (−2.120 to −0.195)	−2.356	0.018 *	5.552	0.018
	Wave vs. Combined	−1.553 (−2.488 to −0.618)	−3.256	0.001 *	10.604	0.001
	Spiral vs. Handwriting	−0.280 (−1.671 to 1.111)	−0.394	0.693	0.155	0.693
	Wave vs. Handwriting	−0.705 (−1.879 to 0.468)	−1.178	0.239	1.387	0.239
	Wave vs. Spiral	−0.407 (−1.904 to 1.090)	−0.533	0.594	0.284	0.594

^a^: The β values were reported on a logit scale. 95% CI: 95% confidence interval. * indicates a significant difference.
