# Supplementary material for: Diagnostic Performance of Artificial Intelligence for Parkinson’s Disease Detection Using Handwriting and Drawing Tasks: A Systematic Review and Meta-Analysis
Source: Sensors (Basel). 2026 Oct 30;26(21):6893. doi: 10.3390/s26216893 (PMC13766507; doi:10.3390/s26216893)
Supplement: Supplementary file 1 [file sensors-26-06893-s001.zip › sensors-4603210-supplementary/Sensors - PRISMA_2020_abstract_checklist-2.pdf]

PRISMA 2020 for Abstracts Checklist

Section and Topic	Item #	Checklist item	Reported (Yes/No)
**TITLE**			
Title	1	Identify the report as a systematic review.	**Yes (Line 2-4)**
**BACKGROUND**			
Objectives	2	Provide an explicit statement of the main objective(s) or question(s) the review addresses.	**Yes (Line 11-14)**
**METHODS**			
Eligibility criteria	3	Specify the inclusion and exclusion criteria for the review.	**Yes (Line 15-18)**
Information sources	4	Specify the information sources (e.g. databases, registers) used to identify studies and the date when each was last searched.	**Yes (Line 14-15)**
Risk of bias	5	Specify the methods used to assess risk of bias in the included studies.	**Yes (Line 19-20)**
Synthesis of results	6	Specify the methods used to present and synthesise results.	**Yes (Line 21-30)**
**RESULTS**			
Included studies	7	Give the total number of included studies and participants and summarise relevant characteristics of studies.	**Yes (Line 30)**
Synthesis of results	8	Present results for main outcomes, preferably indicating the number of included studies and participants for each. If meta-analysis was done, report the summary estimate and confidence/credible interval. If comparing groups, indicate the direction of the effect (i.e. which group is favoured).	**Yes (Line 30-36)**
**DISCUSSION**			
Limitations of evidence	9	Provide a brief summary of the limitations of the evidence included in the review (e.g. study risk of bias, inconsistency and imprecision).	**Yes (Line 37-38)**
Interpretation	10	Provide a general interpretation of the results and important implications.	**Yes (Line 38-41)**
**OTHER**			
Funding	11	Specify the primary source of funding for the review.	**No**
Registration	12	Provide the register name and registration number.	**No**

From: Page MJ, McKenzie JE, Bossuyt PM, Boutron I, Hoffmann TC, Mulrow CD, et al. The PRISMA 2020 statement: an updated guideline for reporting systematic reviews. BMJ 2021;372:n71. doi: 10.1136/bmj.n71. This work is licensed under CC BY 4.0. To view a copy of this license, visit

PRISMA 2020 for Abstracts Checklist

https://creativecommons.org/licenses/by/4.0/
